# Yunvjian-Medicated Serum Protects INS-1 Cells against Glucolipotoxicity-Induced Apoptosis through Autophagic Flux Modulation

**DOI:** 10.1155/2020/8878259

**Published:** 2020-12-14

**Authors:** Caigu He, Xuehua Zheng, Xiuhong Lin, Xinying Chen, Chenyi Shen

**Affiliations:** ^1^Department of Histology and Embryology, Fujian University of Traditional Chinese Medicine, Fuzhou, Fujian 350122, China; ^2^Laboratory of Integration of Traditional and Western Medicine, Fujian University of Traditional Chinese Medicine, Fuzhou, Fujian 350122, China

## Abstract

Yunvjian (YNJ) is a traditional Chinese medicine formula adopted to prevent and treat diabetes. Our previous results from animal experiments showed that YNJ decreased blood glucose. This study aimed to examine the effect of high glucose and high lipid (HG/HL) conditions on the proliferation and apoptosis of INS-1 cells and the possible protective mechanism of YNJ-medicated serum on INS-1 cells exposed to HG/HL conditions. INS-1 cells were cultured in RPMI 1640 medium after being passaged. Then, INS-1 cells in the logarithmic growth phase were collected and divided into five groups: control, HG/HL, HG/HL+5% YNJ-medicated serum, HG/HL+10% YNJ-medicated serum, and HG/HL+20% YNJ-medicated serum. MTT assay and flow cytometry were used to detect proliferation and apoptosis of INS-1 cells, respectively. Protein profiles of INS-1 cells were analyzed using a tandem mass tag (TMT) label-based quantitative proteomic approach. Western blotting was performed to verify the proteomic results. YNJ-medicated serum significantly promoted INS-1 cell proliferation and inhibited apoptosis. Proteomic results from the INS-1 cells in the control, HG/HL, and HG/HL+10% YNJ-medicated serum groups showed that 7,468 proteins were identified, of which 6,423 proteins were quantified. Compared with the HG/HL group,430 differential proteins were upregulated, and 671 were downregulated in the HG/HL+10% YNJ-medicated serum group. Compared with the control group, 711 differential proteins were upregulated and 455 were downregulated in the HG/HL group, whereas 10 differential proteins were upregulated and 9 were downregulated in the HG/HL+10% YNJ-medicated serum group. Furthermore, several proteins related to autophagy, including ATG3, ATG2B, GABARAP, WIPI2, and p62/SQSTM1, were verified by western blotting, and these results were consistent with the results obtained from the proteomics analysis. These results confirmed that the autophagy pathway is critical to glucolipotoxicity in INS-1 cells. YNJ-medicated serum exhibited a protective effect on INS-1 cells cultured under HG/HL conditions by regulating autophagy genes' expression and restoring the autophagic flux.

## 1. Introduction

Autophagy can clear misfolded proteins and damaged organelles produced in cell growth, metabolism, and stress, and timely removal of these substrates is essential for cells to maintain homeostasis and normal physiological activities. Autophagy was first discovered in the 1960s, but the key role and effect of autophagy in physiology and medicine were only recognized after Yoshinori Ohsumi's research in the 1990s. In 2016, the Nobel Prize in Physiology or Medicine was awarded to Yoshinori Ohsumi for these discoveries, bringing autophagy research to new heights. In recent years, studies have found that autophagy is the cell survival mechanism; however, it also participates in the occurrence and development of various diseases, such as tumors, diabetes, liver injury, and leukemia. Autophagy-related signaling pathways as potential therapeutic targets for human diseases have been actively investigated.

The concept of glucolipotoxicity was first proposed by Prentki and Corkey [1]. *β*-cell dysfunction and apoptosis are the two major characteristics of the late stages of Type 2 diabetes mellitus (T2D) [2]. Glucolipotoxicity is an important mechanism underlying the progressive decline in *β*-cell function in patients with T2D. A previous study has indicated that *β*-cells are particularly sensitive to glucolipotoxicity [3]. In the early stages of glucolipotoxicity, the body itself can relieve the glucolipotoxic stress through the compensatory increase in insulin secretion and synthesis; thus symptoms of diabetes are not present. However, with prolonged exposure to glucolipotoxicity, the body's self-compensation function gradually decreases or disappears; then irreversible damage to pancreatic *β*-cell function occurs, including reduced *β*-cell sensitivity to glucose, insufficient insulin secretion, and *β*-cell apoptosis [4], thereby inducing the occurrence of T2D. Thus, glucolipotoxicity mediated *β*-cell loss is a critical causal factor in the late stages of diabetes; however, the exact mechanisms remain unclear.

Several mechanisms have been studied to explain glucolipotoxicity-induced *β*-cell dysfunction and apoptosis, including endoplasmic reticulum (ER) stress, oxidative stress, mitochondrial dysfunction, and autophagy. At present, some researchers believe that diabetes, high glucose (HG), or HG and high lipids (HG/HL) could reduce the level of autophagy in tissues or cells, leading to pathological changes in tissues and cells. These changes can be slowed down by inducing autophagy, which contributes to improving the treatment of diabetes. Antidiabetic drugs such as metformin, rosiglitazone, or GLP-1 receptor agonists have been proven to improve metabolic state by enhancing autophagic activities [5–7]. However, some scholars have held different views and believe that diabetes, HG, or HG/HL can upregulate the expression of autophagy-related proteins and enhance autophagy [8–12].

Yunvjian (YNJ) is a classic traditional Chinese medicine prescription from the Jingyue Quan Shu (complete works of Jingyue), which has a long history in treating diabetes that exerts sound therapeutic effects in clinical practice [13]. YNJ consists of five components as shown in Table 1. The HPLC of YNJ showed that there were 17 characteristic peaks [14]. Previous studies have found that effective components contained in YNJ have apparent effects on lowering blood glucose [15, 16].

This study aimed to examine the effect of HG/HL on the proliferation and apoptosis of INS-1 cells and the possible protective mechanism of YNJ-medicated serum on INS-1 cells exposed to HG/HL conditions.

## 2. Materials and Methods

### 2.1. Animals

A total of 50 SPF male Wistar rats (8-week-old, 180–200 g) were purchased from Shanghai SLAC Laboratory Animal Co., Ltd. (Shanghai, China; license number: SCXK (Shanghai) 2007-0005, certificate number: 0063771) and housed in an SPF barrier system of the Animal Experiment Center of the study university. After adaptive feeding for 1 week, rats were randomly allocated to either the YNJ group (*n* = 30) or the control group (*n* = 20).

### 2.2. Preparation of Normal Saline Serum and YNJ-Medicated Serum

The normal saline serum was drawn from the rats after the normal saline was gavaged into the rats in the control group.

The components and daily dose of YNJ for a 60 kg adult included 15 g of Gypsum Fibrosum (Sheng Shi Gao), 4.5 g of *Anemarrhena asphodeloides* Bunge (Zhi Mu), 30 g of *Rehmannia glutinosa* (Gaertn.) Libosch. ex Fisch. et Mey (Shu Di Huang), 6 g of *Ophiopogon japonicus* (Linn. f.) Ker-Gawl (Mai Dong), and 4.5 g of *Achyranthes bidentata* Blume (Niu Xi). According to body surface area, the daily dose for rats was 6.25 times of an adult human's daily dose. The amount of each type of herb in the YNJ was weighed, and then all of them were placed in a container and soaked in 1,000 mL of water for 1 h. Firstly, we used intense fire to heat the herbs and water until it was boiled and then turned the fire to slow fire for 30 min; the liquid was then poured out to another container. Secondly, we added 500 mL hot water to the first container and then decocted the herbs using intense fire for 15 min; the liquid was also poured out. Thirdly, we mixed the liquid obtained from the first and second steps, which was concentrated on a drug solution containing 1.24 g raw herb per mL, and stored at 4°C.

Rats in the control group were gavaged with normal saline for 6 consecutive days. Rats in the YNJ group were gavaged with YNJ (6.25 g/kg/d) for 6 consecutive days 1 h after the last gavage, rats in both groups were anesthetized with 20% urethane (0.5 ml/100g), and blood was then drawn from the abdominal aorta, which was kept at 4°C for 6 h. After that, the blood was centrifuged at 3500 rpm for 15 min. YNJ-medicated serum and control serum were collected, inactivated at 56°C, filtered with 0.22 *μ*m pore membranes, and stored at −20°C.

### 2.3. Cell Culture and Grouping

INS-1 cells were purchased from Beijing Beina Chuanglian Biotechnology Institute. All experiments were performed using INS-1 cells between passage numbers from 4 to 20. Cells were cultured in RPMI-1640 medium (Gibco 8118248, USA), supplemented with 10% fetal bovine serum (Gibco 8118248, USA), 1% penicillin and streptomycin (Gibco, Grand Island, NY, USA), and 50 *μ*mol/L *β*-mercaptoethanol. Cells were incubated in a humidified atmosphere containing 5% CO_2_ at 37°C and observed by an inverted microscope (×100 and ×200, Nikon, Tokyo, Japan). The medium was renewed once per two days, and cells were passaged once per 6 to 7 days. INS-1 cells in the logarithmic growth phase were used for the following tests. For MTT assay and flow cytometry, INS-1 cells were divided into five groups: (1) control group (continue cultured with RPMI-1640 medium and all supplemented components); (2) HG/HL group (cultured with 25 mmol/L glucose, 0.5 mmol/L palmic acid, and 20% normal saline serum); (3) HG/HL+5% YNJ-medicated serum group (cultured with 25 mmol/L glucose, 0.5 mmol/L palmic acid, 5%YNJ-medicated serum, and 15% normal saline serum); (4) HG/HL+10% YNJ-medicated serum group (cultured with 25 mmol/L glucose, 0.5 mmol/L palmic acid, 10%YNJ-medicated serum, and 10% normal saline serum); and (5) HG/HL+20% YNJ-medicated serum group (cultured with 25 mmol/L glucose, 0.5 mmol/L palmic acid, 20%YNJ-medicated serum). For proteomic and western blotting, INS-1 cells were divided into three groups: (1) control group, (2) HG/HL group, and (3) HG/HL+10% YNJ-medicated serum group.

### 2.4. MTT Assay

To investigate the effect of YNJ-medicated serum on the proliferation rate of INS-1 cells cultured under HG/HL conditions, MTT (3-(4,5-dimethylthiazol-2-yl)-2, 5-diphenyltetrazolium bromide, Roche Life Science (Cat. No. 11465007001, USA)) assay was conducted. In brief, 100 *µ*l of suspension of INS-1 cells (1 × 10^5^ cells/mL) was seeded in each well of 96-well plates and cultured at 37°C in a 5% CO_2_ incubator. After 144 h of culture, INS-1 cells in the logarithmic phase were treated for the five groups accordingly. An equal volume of culture medium was added to the zero-adjustment well. After 48 h of treatment, the first MTT (Roche, 11465007001) was added to the culture medium in each group; after 4 h of incubation, the second MTT was added. The cells were then incubated at 37°C in a 5% CO_2_ incubator overnight, and the absorbance at 590 nm (A590) was determined using a microplate reader (microplate reader, sn:1105002115, Tecan Austria GmbH, Austria).

### 2.5. Flow Cytometry

An Annexin V/propidium iodide (PI) double staining was used to detect cell apoptosis. INS-1 cells in the logarithmic growth phase were collected, digested, and seeded in a 6-well plate at a density of 1 × 10^5^ cells/well with a 2.5 ml culture medium added to each well. INS-1cells were then incubated at 37°C in a 5% CO_2_ incubator. After 144 h of culture, INS-1 cells in the logarithmic phase were treated for the five groups accordingly. The cultural medium of each group was changed every 3 days. After 48 h of treatment, the INS-1 cells were digested with 0.25% trypsin without EDTA, which was stopped by adding a culture solution. After that, INS-1 cells were centrifuged at 1000 rpm/min for 10 min. After discarding the supernatant, 5 × 10^5^ cells were collected after the cells were washed using precooled PBS, which were resuspended in 500 *µ*l binding buffer, and mixed with Annex 5 *µ*l V-FITC. After incubation in the dark for 30 min, 5 *µ*l PI was added and mixed gently. After 5 min incubation in the dark, the proportion of normal cells, necrotic cells, late apoptotic cells, and early apoptotic cells was detected by flow cytometer (BD, USA). A total of 10,000 cells were counted in each group.

### 2.6. Protein Digestion and Peptide Tandem Mass Tag (TMT) Labeling

The sample was sonicated three times on ice using a high-intensity ultrasonic processor (Scientz) in lysis buffer (8 M urea, 1% Protease Inhibitor Cocktail). The remaining debris was removed by centrifugation at 12,000 g at 4°C for 10 min. Finally, the supernatant was collected and the protein concentration was determined with a BCA kit according to the manufacturer's instructions.

For digestion, the protein solution was reduced with 5 mM dithiothreitol for 30 min at 56°C and alkylated with 11 mM iodoacetamide for 15 min at room temperature in the dark. The protein sample was then diluted by adding 100 mM TEAB to urea concentration less than 2 M. Finally, trypsin was added at 1 : 50 trypsin-to-protein mass ratio for the first digestion overnight and 1 : 100 trypsin-to-protein mass ratio for a second 4 h digestion.

After trypsin digestion, the peptide was desalted by Strata X C18 SPE column (Phenomenex) and vacuum-dried. The peptide was reconstituted in 0.5 M TEAB and processed according to the manufacturer's protocol for TMT/10plex kit. Briefly, one unit of TMT reagent was thawed and reconstituted in acetonitrile. The peptide mixtures were then incubated for 2 h at room temperature and pooled, desalted, and dried by vacuum centrifugation.

### 2.7. LC-MS/MS Analysis

The tryptic peptides were fractionated into fractions by high pH reverse-phase HPLC using Agilent 300 Extend C18 column (5 *μ*m particles, 4.6 mm ID, 250 mm length). Briefly, peptides were first separated into 60 fractions with a gradient of 8% to 32% acetonitrile (pH 9.0) over 60 min. Then, the peptides were combined into 18 fractions and dried by vacuum centrifuging.

The tryptic peptides were dissolved in 0.1% formic acid (solvent A) and then directly loaded onto a home-made reversed-phase analytical column (15 cm length, 75 *μ*m i.d.). The gradient comprised an increase of concentration of solvent B (0.1% formic acid in 98% acetonitrile) from 6% to 23% over 26 min, 23% to 35% in 8 min, and climbing to 80% in 3 min and then holding at 80% for the last 3 min, all at a constant flow rate of 400 nL/min on an EASY-nLC 1000 UPLC system.

The peptides were subjected to NSI source followed by tandem mass spectrometry (MS/MS) in Orbitrap Fusion™ Lumos (Thermo) coupled online to the UPLC. The electrospray voltage applied was 2.0 kV. The *m*/*z* scan range was 350 to 1800 for a full scan, and intact peptides were detected in the Orbitrap at a resolution of 70,000. Peptides were then selected for MS/MS using the NCE setting as 28, and the fragments were detected in the Orbitrap at a resolution of 17,500. A data-dependent procedure alternated between one MS scan followed by 20 MS/MS scans with 15.0s dynamic exclusion. Automatic gain control was set at 5E4. The fixed first mass was set as 100 *m*/*z*.

### 2.8. Database Search and TMT Quantification

The resulting MS/MS data were processed using the Maxquant search engine (v.1.5.2.8). Tandem mass spectra were searched against the human database concatenated with the reverse decoy database. Trypsin/P was specified as a cleavage enzyme allowing up to 2 missing cleavages. The mass tolerance for precursor ions was set as 20 ppm in the first search and 5 ppm in the main search, and the mass tolerance for fragment ions was set as 0.02 Da. Carbamidomethyl on Cys was specified as fixed modification and oxidation on Met was specified as variable modifications. FDR was adjusted to <1%, and the minimum score for peptides was set >40.

### 2.9. Bioinformatics Analysis

The classification of differentially expressed proteins (DEPs) was performed based on the annotations obtained from the UniProt-GOA database (http://www.ebi.ac.uk/GOA/). Proteins were classified by Gene Ontology (GO) annotation into three categories: biological process (BP), cellular component (CC), and molecular function (MF). Encyclopedia of Genes and Genomes (KEGG) database was used to identify enriched pathways by a two-tailed Fisher's exact test to test the enrichment of DEPs against all identified proteins. The pathway with a corrected *p* value <0.05 was considered significant. These pathways were classified into hierarchical categories according to the KEGG website (http://www.genome.jp/kegg/). For further hierarchical clustering based on different protein functional classifications, we first collated all the categories obtained after enrichment along with their *p* values and then filtered for those categories, which were at least enriched in one of the clusters with *p* values <0.05. This filtered *p* value matrix was transformed by the function *x* = −log10 (*p* value). Finally, these *x* values were *z*-transformed for each functional category. These *z* scores were then clustered by one-way hierarchical clustering (Euclidean distance, average linkage clustering) in Genesis.

### 2.10. Western Blotting Analysis

Proteins were separated by SDS-PAGE and transferred to a PVDF membrane. The membranes were blocked with 5% BSA in 1x Tris-buffer containing 0.1% Tween-20 for 1 h. The blots were incubated overnight with primary antibodies, including ATG3 (Abcam, ab108251, 1 : 1000), ATG2B (RayBiotech, 144-o8498-5, 1 : 1000), P62/SQSTM1 (Abcam ab91526, 1 : 1000), WIPI2 (Abcam ab229225, 1 : 1000), GABARAP (Abcam, ab109299, 1 : 1000), and *β*-actin (Santa Cruz Biotechnology, 1 : 1000), and they were visualized with anti-rabbit horseradish peroxidase-conjugated secondary antibody (Vector Laboratories, Burlingame, 1 : 10000) for 1 h. The blots were developed with ECL chemiluminescence detection reagent (Bio-Rad, CA, USA).

### 2.11. Statistical Analysis

Statistical analyses were performed using IBM SPSS 24.0 statistical software (IBM Corp, USA). Measurement data were presented as mean ± standard deviation (SD). Two-way analysis of variance (ANOVA) was used for between-group comparisons of the expression of proteins. A *p* value less than 0.05 was considered a significant difference.

## 3. Results

### 3.1. Effect of YNJ-Medicated Serum on the Proliferation of INS-1 Cells under HG/HL Conditions

Different concentrations of YNJ-medicated serum exhibit different effects on the proliferation of INS-1 cultured under HG/HL condition for 48 h. The A590 values in the control, HG/HL, HG/HL+5%, HG/HL+10%, and HG/HL+20% YNJ-medicated serum groups were 2.38 ± 0.17, 1.75 ± 0.32, 2.11 ± 0.09, 2.35 ± 0.16, and 2.09 ± 0.13, respectively. There were statistically significant differences in the A590 values between the control and HG/HL groups (*p* < 0.01), HG/HL and HG/HL+5%YNJ-medicated serum groups (*p* < 0.05), HG/HL and HG/HL+10 %YNJ-medicated serum groups (*p* < 0.01), and HG/HL and HG/HL+20 %YNJ-medicated serum groups (*p* < 0.05). The results suggested that HG/HL could reduce cell proliferation after 48 h of culture. All 5%, 10%, and 20% YNJ-medicated serum promoted the proliferation of INS-1 cells cultured under HG/HL conditions, and 10% concentration of YNJ-medicated serum had a better effect than 5% and 20% concentration of YNJ-medicated serum (Table 2).

### 3.2. Effect of YNJ-Medicated Serum on the Apoptotic Rates of INS-1 Cells under HG/HL Conditions

**Figure 1:**
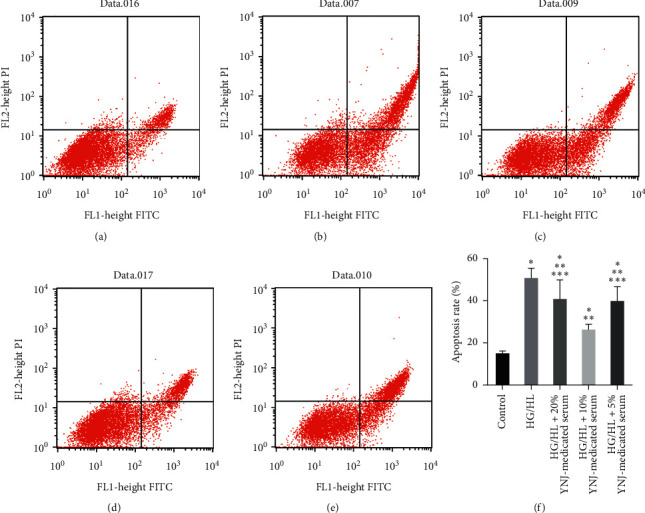
The effect of YNJ-medicated serum on INS-1 cell apoptosis detected by Annexin V-FITC/PI staining. (a) Control group. (b) HG/HL group. (c) HG/HL +20% YNJ-medicated serum group. (d) HG/HL +10% YNJ-medicated serum group. (e) HG/HL +5% YNJ-medicated serum group. (f) Results based on the two-way ANOVA: compared with the control group, ^*∗*^*p* < 0.01; compared with the HG/HL group, ^*∗∗*^*p* < 0.05; compared with the 10% YNJ-medicated serum group, ^*∗∗∗*^*p* < 0.05.

Different concentrations of YNJ-medicated serum exhibited different effects on the apoptosis of INS-1 cells cultured under HG/HL conditions for 48 h (Figure 1). The apoptosis rate of INS-1 cells in the control group was significantly lower than that in the HG/HL group (*p* < 0.01), the apoptosis rate of INS-1 cells in the HG/HL group was significantly higher than that in the HG/HL+5% YNJ-medicated serum group (*p* < 0.05), HG/HL+10% YNJ-medicated serum group (*p* < 0.01), and HG/HL+20% YNJ-medicated serum group (*p* < 0.05), and significant differences were also observed between HG/HL+5% and HG/HL+10% YNJ-medicated serum groups (*p* < 0.01), as well as between HG/HL+10% and HG/HL+20% YNJ-medicated serum groups (*p* < 0.01). These results indicated that HG/HL could increase INS-1 cell apoptosis after 48 h of culture, and YNJ-medicated serum inhibited INS-1 cell apoptosis. The inhibitory effect of 10% YNJ-medicated serum on INS-1 cell apoptosis was superior to 5% and 20% YNJ-medicated serum.

### 3.3. TMT-Based Proteomic Isobaric Multiplex Labeling and LC-MS/MS Analysis

**Figure 2:**
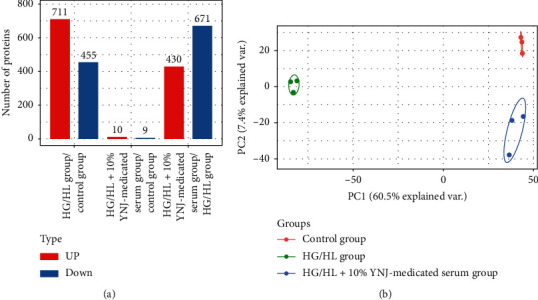
The results of differentially expressed proteins and sample repeatability tests in three groups. (a) Histogram of the number distribution of differentially expressed proteins: 10 differential proteins were upregulated and 9 were downregulated in the HG/HL+10% YNJ-medicated serum group when compared with the control group; 430 differential proteins were upregulated and 671 were downregulated in the HG/HL+10% YNJ-medicated serum group when compared with the HG/HL group; 711 differential proteins were upregulated and 455 were downregulated in the HG/HL group when compared with the control group. (b) Two-dimensional scatter plot of principal component analysis (PCA) distribution of three samples using quantified proteins. Three biological replicates were performed in this study.

The proteomics was performed on the INS-1 cells in the following three groups: control, HG/HL, and HG/HL+10% YNJ-medicated serum groups. In this study, we used TMT labeling, LC-MS/MS analysis, and bioinformatics analysis to compare the complete proteomic profiles of INS-1 cells in the three groups. A total of 7,468 proteins from INS-1 cells in three biological replicates were identified, and 6,423 proteins were quantifiable. Among those proteins, 430 differential proteins were upregulated and 671 were downregulated in the HG/HL+10% YNJ-medicated serum group compared with the HG/HL group. Compared with the control group, 711 differential proteins were upregulated and 455 were downregulated in the HG/HL group, whereas 10 differential proteins were upregulated and 9 were downregulated in the HG/HL+10% YNJ-medicated serum group. All these data were shown in Figure 2(a). The quantification reproducibility was also investigated; as shown in Figure 2(b), we found that the three quantitative experiments were highly reproducible.

### 3.4. Functional Analysis of Significant DEPs in INS-1 Cells

**Figure 3:**
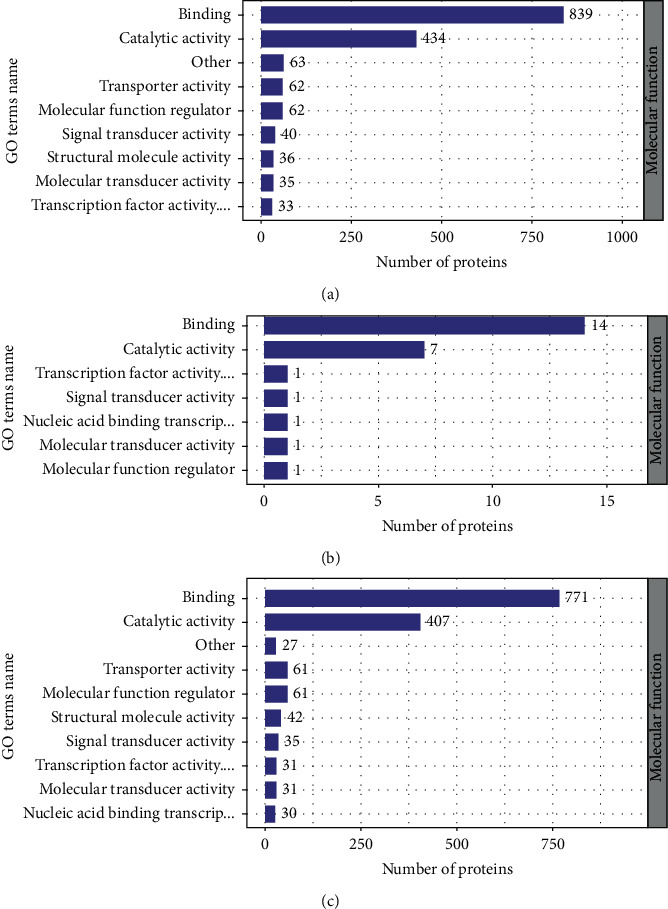
Molecular function statistical distribution chart of differentially expressed proteins under each GO category (2^nd^ Level). (a) 54.2% protein related to binding and 28.0% to catalytic activity in the HG/HL group compared with the control group. (b) 53.8% protein related to binding and 26.9% to catalytic activity in the HG/HL+10% YNJ-medicated serum group compared with the control group. (c) 52.5% protein related to binding and 27.7% to catalytic activity in the HG/HL +10% YNJ-medicated serum group compared with the HG/HL group.

To show the differences between up- and downregulated expressed proteins and analyze their biological functions and subcellular localization, functional enrichment and enrichment-based clustering of those DEPs were performed. The subcellular localization of significantly DEPs was characterized (Table 3). After that, we analyzed the molecular functions of DEPs (Figure 3). Most proteins participated in binding and catalytic activity. These results suggested that DEPs could have a broad localization distribution and biological functions.

### 3.5. Functional Enrichment Analysis of DEPs

**Figure 4:**
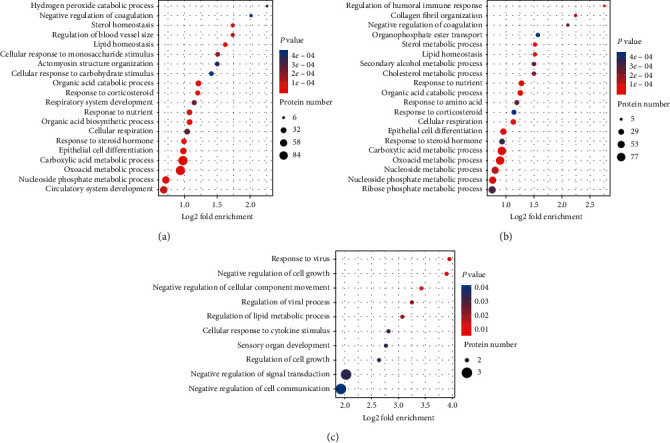
GO (Gene Ontology) enrichment bubble plot of differentially expressed proteins in the biological process. (a) There were hundreds of different proteins in the HG/HL group compared with the control group, and most of them were related to the metabolic process. (b) There were also hundreds of different proteins in the HG/HL+10% YNJ-medicated serum group compared with the HG/HL group, and most of them were also related to the metabolic process. (c) There were only 22 different proteins in the HG/HL +10% YNJ-medicated serum group compared with the control group, with three related to cell transduction, three related to cell communication, and two associated with the regulation of lipid metabolic process. *p* < 0.05.

Based on the annotations of all the identified proteins and the screening of DEPs, we conducted a functional enrichment analysis of these DEPs. GO, KEGG pathway, and protein domain enrichment analyses were used to explore whether specific functional categories of DEPs tended to be significantly enriched. *p* value was calculated using Fisher's exact test for each pathway and functional category. Functional categories and pathways that were significantly enriched were plotted in the bubble chart (Figure 4).

**Figure 5:**
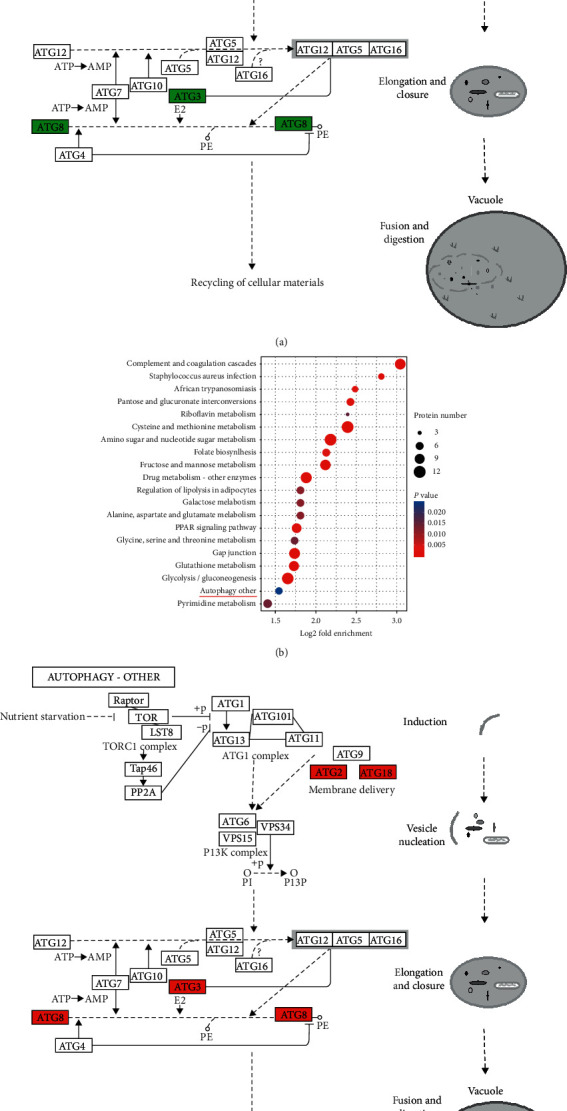
KEGG pathway enrichment showing significant enrichment of differentially expressed genes in signaling pathways. (a) Downregulated differentially expressed genes in the autophagy pathway in the HG/HL group compared with the control group. (b, c) Upregulated differentially expressed genes in the autophagy pathway in the HG/HL+10% YNJ-medicated serum group compared with the HG/HL group. The red color represents that the protein was upregulated, and the green color represents that the protein was downregulated.

To reveal the nature of DEPs, KEGG pathway enrichment analysis was carried out, which showed that the upregulated proteins were mainly enriched in 20 pathway entries, and the autophagy pathway and metabolism pathways (riboflavin metabolism, cysteine and methionine metabolism, amino sugar and nucleotide sugar metabolism, fructose and mannose metabolism, galactose metabolism, alanine, aspartate and glutamate metabolism, glycine, serine and threonine metabolism, glutathione metabolism, and pyrimidine metabolism) were perhaps the most relevant pathways (Figures 5).

### 3.6. Verification of DEPs by Western Blotting Analysis

**Figure 6:**
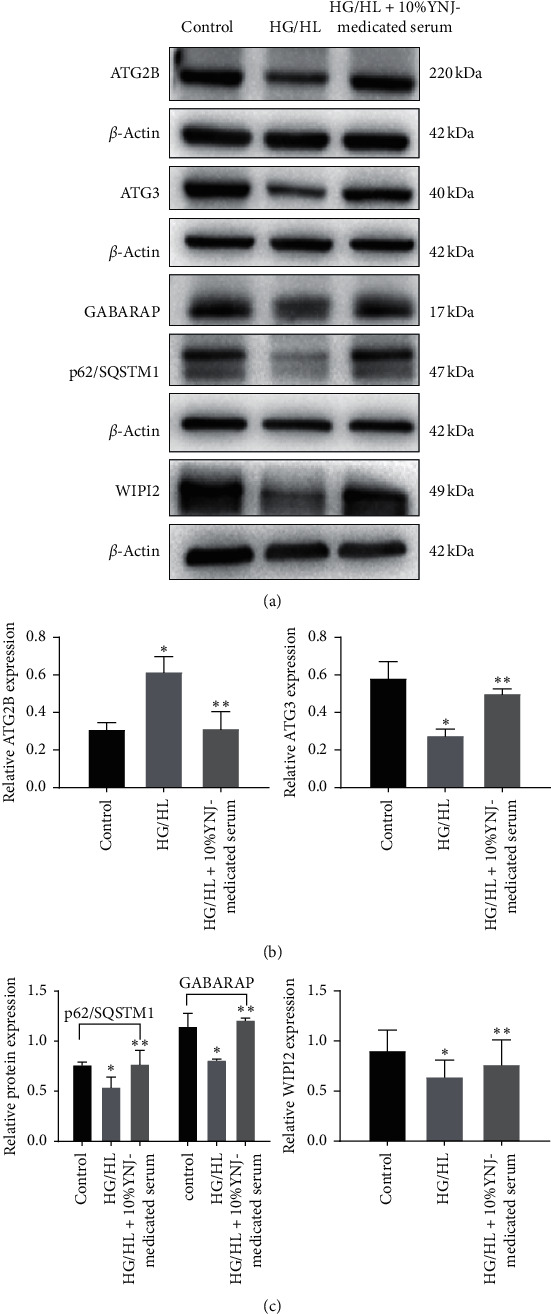
Effects of 10% YNJ-medicated serum on the expression of ATG2B, ATG3, WIPI2, p62/SQSTM1, and GABARAP protein in INS-1 cells exposed to HG/HL conditions. (a) Representative western blots showing protein expression. (b) Quantitative analysis of protein. Bar graphs represent the means ± SD from three independent experiments. Compared with the control group, ^*∗*^*p* < 0.05; compared with the HG/HL group, ^*∗∗*^*p* < 0.05.

To validate DEPs identified by the quantitative proteomic analysis, western blotting was adopted to detect the expression levels of autophagy-related 2B (ATG2B), sequestosome-1 (P62/SQSTM1), Gamma-aminobutyric acid receptor-associated protein (GABARAP), WD repeat domain phosphoinositide-interacting protein 2 (WIPI2), and ubiquitin-like-conjugating enzyme ATG3 (ATG3) (Table 4). Results showed that compared with the control group, the expression levels of these five proteins were downregulated in the HG/HL group, while the inhibitory effect of HG/HL on the expression of all these proteins was partly reversed by the treatment using 10% YNJ-medicated serum (Figure 6). These results were similar to the protein expression levels observed from the iTRAQ approach, implying the proteomic analysis's reliability.

## 4. Discussion

Autophagy is the process that delivers cytoplasmic material to the lysosome for degradation. According to different delivery routes from the cytoplasmic material to the lysosomal lumen, autophagy is mainly divided into three types: chaperone-mediated autophagy, microautophagy, and macroautophagy [17]. Among the three types of autophagy, macroautophagy is an essential mechanism for eukaryotic cells to maintain the internal environment's stability and organ function, which is often referred to as autophagy. All these types of autophagy are related to hormone-secreting cells, such as pancreatic *β*-cells [18–20].

Autophagy is a highly conserved process and is governed by a series of genes. So far, more than 30 autophagy-related genes have been identified. The autophagy process can be divided into three steps: (1) initiation/nucleation (formation of phagophore), (2) expansion/completion (formation of autophagosome), and (3) degradation/retrieval (formation of autolysosome). Different autophagy-related genes are involved in different steps [21, 22]. The WIPI2 (a PI3P-binding effector protein) and ATG2-GABARAP/GABARAP-L1 are involved in the formation and nucleation of the isolation membrane. The extension and completion of the autophagosome membrane are related to two ubiquitin-like systems, ATG12 and ATG8. ATG8 is a ubiquitin-like protein that can bind to ATG3 via ATG7, and LC3-ATG3 can bind to the ATG12 system, which is then localized to the isolation membrane. In addition, PI3P-WIPI2-ATG2 on the ER membrane translocates to LC3-II-positive autophagosomes and participates in the early extension of autophagosome membranes. After autophagosome formation, the autophagosome fuses with lysosomes to degrade its contents.

Autophagy was first believed to be nonselective. However, recent studies have found that autophagy can selectively remove protein aggregates and damaged organelles in cells through receptor proteins [23–27]. Selective autophagy usually requires the presence of some adaptor proteins. p62/SQSTM1 was the first selective autophagy adaptor protein discovered in mammals, which have ubiquitin-binding domains and LC3-interacting region so that these proteins can bind to ubiquitinated proteins and transport them to autophagosomes [28]. Meanwhile, p62/SQSTM1 undergoes self-oligomerization through its N-terminal PB1 domain, which finally enters the autophagolysosome, leading to degradation of the ubiquitinated substrate [29–31]. As an important selective autophagy adaptor protein, p62/SQSTM1 plays a role in the clearance of ubiquitinated proteins [32].

In this present study, the MTT assay result showed that HG/HL reduced, whereas the YNJ-medicated serum increased the proliferation of INS-1 cells. Flow cytometry results showed that HG/HL increased, whereas the YNJ-medicated serum decreased the apoptosis of INS-1 cells. Our results were consistent with previous study findings [33–38]. For instance, Masini et al. [33, 34] found that mice with *β*-cell-specific deletion of autophagy-related gene ATG7 had impaired glucose tolerance, increased *β*-cell apoptosis, and decreased *β*-cell division and proliferation, resulting in a reduction in the number of *β*-cells and insulin secretory. Cnop et al. reported that palmitate and high glucose inhibited gene expression related to autophagy and lysosomal function [35]. Other studies using pH-sensitive LC3 expression vectors and measuring p62 degradation showed that palmitate and high glucose inhibited autophagosome-lysosome fusion and lysosome acidification [36–38].

**Figure 7:**
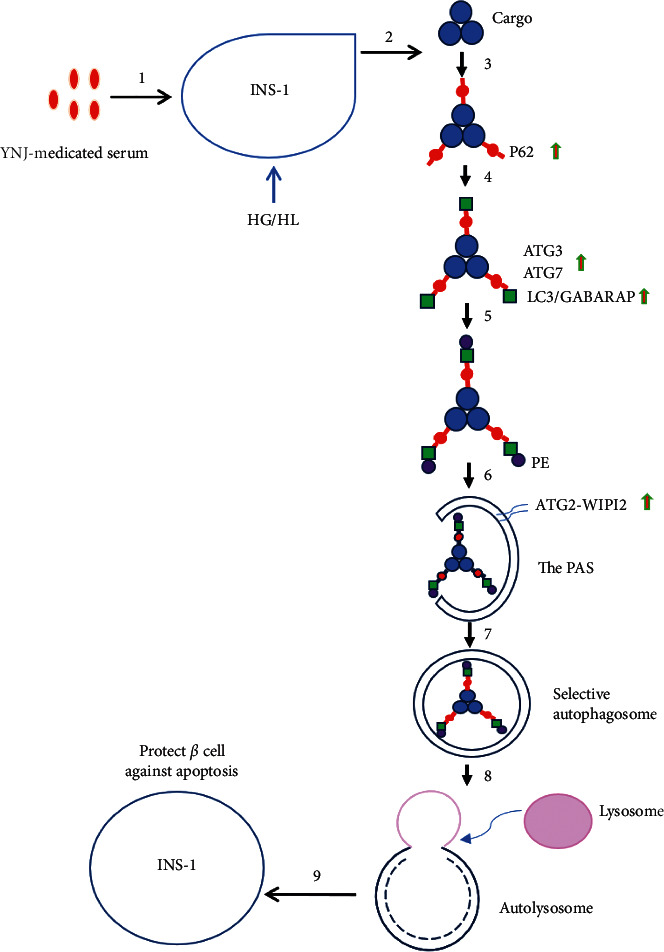
The possible mechanism of YNJ-medicated serum on the regulation of autophagy in INS-1 cells. 1: treatment of INS-1 cells cultured under HG/HF conditions with YNJ-medicated serum. 2: there were some damaged organelles and proteins in INS-1 cells. 3: damaged organelles or proteins binding to p62. 4: p62 binding to LC3/GABARAP with the involvement of ATG3 \ATG7. 5: LC3 binding to PE to form LC3-II;. 6: LC3-II; and ATG2-WIPI2 were localized in PAS. 7: with the extension of the membrane, autophagosomes were formed. 8: autophagosomes fused with lysosomes to form autolysosomes where the sequestered contents were degraded. 9: After the clearance of damaged substances, INS-1 cells were protected from HG/HF-induced apoptosis by YNJ-medicated serum.

In the present study, the proteomic results showed that HG/HL reduced the expression levels of ATG2B, WIPI2, ATG3, GABARAP, and p62/SQSTM1 in INS-1 cells, whereas the YNJ-medicated serum increased the expression of these proteins in INS-1 cells cultured under HG/HL condition, with expression levels similar to those in the control group. Our results from western blot analysis also confirmed these findings. Therefore, our findings suggested that HG/HL reduced the autophagy levels in INS-1 cells, while YNJ-medicated serum increased the expression of autophagy-related genes, especially the expression of genes related to autophagy initiation and autophagosome formation, and thus increased the level of autophagy. The YNJ-medicated serum can clear damaged proteins and organelles through autophagy, thereby achieving an inhibitory effect on the apoptosis of INS-1 cells. The possible mechanism of YNJ-medicated serum in autophagy regulation in INS-1 cells is shown in Figure 7 [39].

While there were contradictory findings with regard to the relationship between p62/SQSTM1 and autophagy activity in the literature [35, 38, 40–43], our results supported other researchers' findings in that the increase in p62/SQSTM1 levels indicates the inhibition of autophagy activity [35, 38, 40, 41]. The increased p62/SQSTM1 levels were also observed in the islets of human T2D patients [44]. In this study, we also found a similar phenomenon in the INS-1 cells, and the reason may be that p62/SQSTM1 serves as a stress protein; its expression levels will be greatly increased under stress conditions such as HG/HL. Due to the complex relationship between p62/SQSTM1 and autophagy, as well as the highly dynamic and multistage process of cellular autophagy itself, when we determine changes in autophagic activity in cells, we must evaluate the changes in autophagic flux, that is, the dynamic changes in the formation of autophagosome, the fusion of autophagosomes with lysosomes, and substrate degradation.

With regard to the relationship between diabetes, HG, or HG/HL conditions and the autophagic activities, there were three types of results from in vivo and in vitro studies: most researchers believe that diabetes, HG, or HG/HL conditions can reduce the level of autophagy, thus triggering pathological changes in tissues and cells; some believe that diabetes, HG, or HG/HL can upregulate the expression of autophagy-related proteins and enhance autophagy [8], and others believe that autophagy plays a dual role in diabetes, which may be related to different HG environments, different intervention methods, and different types of diabetes. These inconsistent findings might be due to differences in experimental methods, models, time of action, and sample sizes. Our study findings supported most researchers' findings that HG/HL could reduce the level of autophagy, and like metformin [5], the first-line drug for the treatment of diabetes, and other drugs such as rosiglitazone and glucagon-like peptide 1 (GLP-1) [5–7], YNJ-medicated serum was shown to enhance the autophagy of INS-1 cells in the present study, which indicates that YNJ's therapeutic effect on diabetic patients is related to its enhancement of autophagy in INS-1 cells.

## 5. Conclusions

This was the first study demonstrating the protective mechanism of YNJ-medicated serum in INS-1 cells against glucolipotoxicity-induced apoptosis. Overall, 10% YNJ-medicated serum increased the proliferation and reduced the apoptosis of the INS-1 cells exposed to HG/HL conditions, with the mechanism of increasing the expression of the autophagic genes and enhancing the autophagic activities. The mechanism for the use of YNJ to treat diabetes was related to the increase of the proliferation and reduction of the apoptosis of the INS-1 cells through the regulation of the autophagic flux.

## Figures and Tables

**Table 1:** Components of YNJ.

No.	Names of YNJ components (Chinese Pin Yin, Chinese character)	Family the component belongs to
1	Gypsum Fibrosum (Sheng Shi Gao, 生石膏)	Mineral (CaSO_4_·2H_2_O)
2	*Anemarrhena asphodeloides* Bunge (Zhi Mu, 知母)	Liliaceae
3	*Rehmannia glutinosa* (Gaetn.) Libosch. ex Fisch. et Mey (Shu Di Huang, 熟地黄)	Scrophulariaceae
4	*Ophiopogon japonicus* (Linn. f.) Ker-Gawl (Mai Dong, 麦冬)	Liliaceae
5	*Achyranthes bidentata* Blume (Niu Xi, 牛膝)	Amaranthaceae

**Table 2:** The effect of different concentrations of YNJ-medicated serum on the proliferation of INS-1 cells cultured under high glucose and high lipid (HG/HL) conditions (mean ± SD).

Group	*n*	Absorbance at 590 nm (A_590_)	95% confidence interval	
			Lower limit	Upper limit
Control group	3	2.38 ± 0.17	2.17	2.60
HG/HL group	3	1.75 ± 0.32^*∗*^	1.53	1.96
HG/HL+5% YNJ-medicated serum group	3	2.11 ± 0.09^△^	1.90	2.33
HG/HL+10% YNJ-medicated serum group	3	2.35 ± 0.16^△^	2.14	2.57
HG/HL+20% YNJ-medicated serum group	3	2.09 ± 0.13^△^	1.87	2.30

INS-1 cells in the logarithmic growth phase were divided into five groups: (1) control group (continue cultured with RPMI-1640 medium and all supplemented components); (2) HG/HL group (cultured with 25 mmol/L glucose, 0.5 mmol/L palmic acid, and 20% normal saline serum); (3) HG/HL+5% YNJ-medicated serum group (cultured with 25 mmol/L glucose, 0.5 mmol/L palmic acid, 5%YNJ-medicated serum, and 15% normal saline serum); (4) HG/HL+10% YNJ-medicated serum group (cultured with 25 mmol/L glucose, 0.5 mmol/L palmic acid, 10% YNJ-medicated serum, and 10% normal saline serum); and (5) HG/HL+20% YNJ-medicated serum group (cultured with 25 mmol/L glucose, 0.5 mmol/L palmic acid, 20%YNJ-medicated serum). MTT assay was used to detect the proliferation of INS-1 cells after 48 h of culture. Compared with the control group, ^*∗*^*p* < 0.01; compared with the HG/HL group, ^△^*p* < 0.05.

**Table 3:** The subcellular location of up- and downregulated proteins (HG/HL+10% YNJ-medicated serum group versus HG/HL group).

Subcellular location	Upregulated proteins (%)	Downregulated proteins (%)
Nucleus	22.56	35.62
Cytosol	44.19	15.8
Mitochondria	9.07	14.9
Extracellular	12.79	13.86
Plasma membrane	3.72	13.41
Other	1.63	6.41
Cytoplasm, nucleus	3.72	—
Cytoskeleton	2.33	—

**Table 4:** Differently expressed protein verified by western blotting in iTRAQ.

Protein name	Protein accession	HG/HL+10% YNJ-medicated serum group/control group ratio	*p* value	HG/HL group/control group ratio	*p* value	HG/HL+10% YNJ-medicated serum group/HG/HL group ratio	*p* value
p62/SQSTM1	O08623	0.986	0.607	0.700	0.022^*∗*^	1.409	0.004^*∗∗*^
GABARAP	P60517	0.996	0.998	0.697	0.030^*∗*^	1.429	0.016^*∗*^
ATG2B	F1MAF8	0.914	0.243	0.573	0.001^*∗∗*^	1.596	0.001^*∗∗*^
WIPI2	Q6AY57	1.048	0.535	0.753	0.025^*∗*^	1.392	0.003^*∗∗*^
ATG3	Q6AZ50	0.995	0.930	0.640	0.005^*∗∗*^	1.555	0.005^*∗∗*^

^*∗*^ *p* < 0.05 and ^*∗∗*^*p* > 0.01.

## Data Availability

The data used to support the findings of this study are available from the corresponding author upon request.
